# A Wearable Sensor System for Physical Ergonomics Interventions Using Haptic Feedback

**DOI:** 10.3390/s20216010

**Published:** 2020-10-23

**Authors:** Carl Mikael Lind, Jose Antonio Diaz-Olivares, Kaj Lindecrantz, Jörgen Eklund

**Affiliations:** 1 Division of Ergonomics, School of Engineering Sciences in Chemistry, Biotechnology and Health, KTH Royal Institute of Technology, Hälsovägen 11C, 14157 Huddinge, Sweden; jadiaz@kth.se (J.A.D.-O.); kajli@kth.se (K.L.); jorekl@kth.se (J.E.); 2 Unit of Occupational Medicine, Institute of Environmental Medicine, Karolinska Institutet, Solnavägen 4, 11365 Stockholm, Sweden; 3 Department of Biosystems, Biosystems Technology Cluster Campus Geel, KU Leuven, Kleinhoefstraat 4, 2440 Geel, Belgium; 4 Science Park Borås, University of Borås, SE-501 90 Borås, Sweden

**Keywords:** smart workwear system, risk assessment, prevention, work technique training, wearable sensors, inertial measurement units, workwear, vibrotactile feedback, musculoskeletal disorders, work postures

## Abstract

Work-related musculoskeletal disorders are a major concern globally affecting societies, companies, and individuals. To address this, a new sensor-based system is presented: the Smart Workwear System, aimed at facilitating preventive measures by supporting risk assessments, work design, and work technique training. The system has a module-based platform that enables flexibility of sensor-type utilization, depending on the specific application. A module of the Smart Workwear System that utilizes haptic feedback for work technique training is further presented and evaluated in simulated mail sorting on sixteen novice participants for its potential to reduce adverse arm movements and postures in repetitive manual handling. Upper-arm postures were recorded, using an inertial measurement unit (IMU), perceived pain/discomfort with the Borg CR10-scale, and user experience with a semi-structured interview. This study shows that the use of haptic feedback for work technique training has the potential to significantly reduce the time in adverse upper-arm postures after short periods of training. The haptic feedback was experienced positive and usable by the participants and was effective in supporting learning of how to improve postures and movements. It is concluded that this type of sensorized system, using haptic feedback training, is promising for the future, especially when organizations are introducing newly employed staff, when teaching ergonomics to employees in physically demanding jobs, and when performing ergonomics interventions.

## 1. Introduction

### 1.1. Background and Problem Description: Musculoskeletal Disorder and Risk Assessments

Work-related diseases and disorders constitute a large problem globally and affect societies, organizations, and individuals. The attributed costs have been estimated as 3.9% of the Gross Domestic Product globally and 3.3% within the EU [1]. Musculoskeletal disorders (MSDs) are the most common work-related health disorder, constituting 40% of the global compensation costs of occupational and work-related accidents and diseases [2]. Despite a large focus on increasing automation in the handling of goods, the exposure to major work-related MSD risk factors such as heavy and repetitive manual handling and awkward postures [3,4,5,6,7] remains frequent in the working population [8]. International surveys indicate that the amount of manual handling activities has not declined substantially in the last decades [8,9], although an increasing number of industrial processes for goods handling have been automated. Manual handling is still a feasible solution in many situations due to its high flexibility and low investment costs compared with many fully or semi-automated solutions [10,11]. Therefore, future manufacturing systems, such as Industry 4.0 [12], which has increased utilization of automated processes, may partly rely on the manual handling of goods for the foreseeable future. When manual handling cannot be avoided, employers are obligated to perform a risk assessment to ensure that operations can be performed without adverse health effects [13]. However, risk assessments are often initiated late in the process and often initiated reactively from employees’ reports of symptoms, disorders or disabilities [14]. Such reactive risk-management strategies may result in unnecessary adverse physical loads due to workstations or jobs with a poor fit to the employees’ anthropometrics (e.g., strength, stature, and reach), as well as mental abilities. As a result, poor work design may induce large direct and indirect costs for the organizations related to increased quality deficiency rates, for example [15,16,17,18].

An alternative is to shift the focus to proactive risk management [19], where risk assessments of potential hazards are performed at earlier stages before symptoms such as work-related pain have emerged [20,21,22,23]. Such risk assessments are often performed by professional ergonomists with the direct or indirect support of observation-based assessment tools [14,24,25]. Despite the broad applicability of observation-based assessment tools [26,27], they can have low precision and reliability [28,29], and can additionally be less cost-efficient than technical measurement instruments when considering the precision of the obtained data [30]. To compensate for such disadvantages, the assessment of risk factors for MSDs by using observation-based assessment tools may benefit from being complemented with increased use of technical measurement instruments [31], such as sensors.

### 1.2. State-of-the-Art Sensor-Based Solutions in Risk Assessments and Work Technique Training

Technical measurement instruments for the recording of work postures and movements have in the past mainly been applicable for research, and they are still scarcely used among professional ergonomists compared to observation-based tools [24]. For example, among professional ergonomists in the US, only about 12% reported using non-optical motion capture instruments at least once every six months, and less than 5% reported using electronic goniometer for the wrist or trunk at least once every six months. Recent rapid technological developments have resulted in an increasing number of low-cost solutions for the monitoring of human movements and postures [32,33,34,35,36]. While some technical solutions solely focus on single joints [37], others target multiple body segments, e.g., the trunk, arms, hands, and the lower limbs [38,39]. Some of these solutions are stationary [39,40], making them less suitable for monitoring tasks where the monitored employee is not working in a fixed work area, when compared to ambulatory systems [38,41]. Given the nature of many jobs, ambulatory solutions may be more applicable for monitoring a larger variety of tasks.

The majority of these sensor-based technical solutions provide kinematics data or perform classification of the type of physical activity [42,43], but only a few have integrated a system for automatic mapping of the kinematics data to research-based risk level metrics that can be used for occupational applications for the risk assessment of physical workloads or MSDs risk factors [44,45,46,47].

In the handful of existing systems, the risk assessment usually includes mapping of the kinematics data to criteria derived from research-based observation tools, e.g., the Strain Index [48,49], the Ovako Working Posture Assessment System (OWAS) [50,51,52], the Rapid Upper Limb Assessment (RULA) [45,52,53,54], the Revised NIOSH lifting equation [52,55,56], or a single criterion associated with, for example, the maximal acceptable levels of energy expenditure to avoid global (whole-body) fatigue [44], or a criterion based on threshold values associated with an increased risk of developing MSDs based on epidemiological studies [47]. The risk assessment may give useful information that can be used for the guidance of measures and evaluation of the effectiveness of previously implemented measures [14]. In addition to providing risk assessments, some of these systems also enable real-time analysis that can give feedback to the wearer for work technique training [45,57].

For example, Vignais et al. [45] evaluated the effect of real-time visual feedback provided via a transparent head-mounted display to reduce postural load in a simulated industrial manual handling task, using the RULA-method score as criteria. When compared to the control group, the participants provided with real-time feedback spent significantly less time in the high exposure categories derived from the RULA score. The study suggests that real-time feedback may be an effective strategy to alter the work technique, at least in the short term. However, visual feedback may be unsuitable for work tasks requiring continuous visual control.

The current evidence does not provide strong support that interventions targeting the individuals’ work technique by, for example, education training on body mechanics and back care, as well as lifting techniques training, are effective in reducing musculoskeletal disorders [58,59,60]. Still, there is a growing number of studies indicating that the use of extrinsic (augmented) direct-feedback by using auditory, visual, or haptic displays [41] can be effective in reducing biomechanical exposures in manual handling [45,61,62], in computer work [63], or in surveillance operations [64].

The traditional work technique training uses trained instructors and is, therefore, resource intensive. Biofeedback training is effective in terms of conveying the message, and it is also efficient since self-training is involved to a large extent. There is consequently the potential to apply extrinsic direct-feedback training in combination with cost-efficient monitoring of workers for assessing risks associated with physical loads. This type of technology needs to be adaptive for monitoring a broad range of risk factors and easy to use; additionally, it should not interfere with the wearer and the tasks to be performed. Such a system would require a platform capable of recording, storing, and presenting workload-related data continuously for a period up to a full working day and unobtrusively in various types of workplaces, e.g., manufacturing industry, building sites, nursing homes, etc.

Just as work technique training has relied on trained instructors, feedback training in sport has typically depended on personal coaching. With the development in recent years of wearable movement sensors, for instance, inertial measurement units (IMU) incorporated in clothing, some examples of support in sports have emerged. These systems typically lack real-time feedback and there is still a clear emphasis on capturing data for analysis performed in hindsight, and the feedback is often limited to visual presentations in graphical form on a screen [65,66,67]. One exception is a pilot study on locomotion training where real-time haptic feedback was introduced [68]. Here vibrotactile actuators were used for immediate feedback on cadence and foot-drop angle during running.

### 1.3. New Sensor-Based Solution for Risk Assessment and Work Technique Training: The Smart Workwear System

The Smart Workwear System is an ambulatory and flexible sensor-based technical solution targeting work-related physical and psychosocial stressors that can have adverse or promoting effects on health and direct or indirect effects on work performance. Depending on the targeted exposure type, suitable sensors can be used, including the following: accelerometer-based sensors, IMU sensors, or strain sensors for monitoring human movement and postures [69]; electrocardiogram and thoracic electrical bio-impedance sensors for monitoring the heart rate, respiratory rates, energy expenditure, or heart rate variability [44]; pressure sensors for monitoring force exertions; electromyography (EMG) sensors for monitoring muscle activity levels, muscle rest (gaps), muscle activity variability, or muscle fatigue. This diversity makes the system usable for a large variety of applications, and previous applications where the system has been tested include healthcare, industrial manufacturing, construction, office work, service, and transport, with tasks ranging from driving, computer work, postal delivery, and assembly operations, to order picking and cleaning [44,47,69,70,71]. To prevent work-related MSDs (WMSDs), the system can be used for exposure measurements, risk assessments, work design, and training of workers.

**Figure 1:**
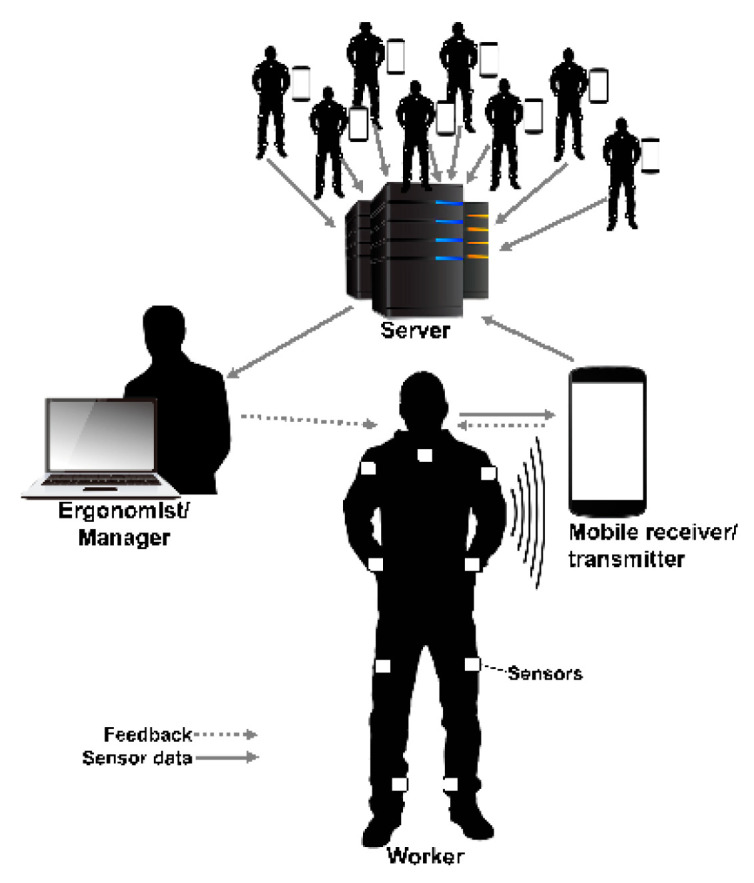
Overview of the Smart Workwear System, illustrating information flow from workwear-worn sensors to a mobile device such as a Smartphone, which can provide feedback directly to the worker via, e.g., auditory, visual, or haptic feedback, or send the information to a local server for real-time assessment or post-analysis of single or multiple workers, based on Lind et al. [47].

As an illustration of an application of the Smart Workwear System (Figure 1), body-worn sensors collect kinematic data on human movements that are sent to a receiver, such as a smartphone, for storage and real-time analysis, using mobile computing software (ErgoRiskLogger). Raw or preprocessed data can additionally be sent to a server, allowing for storage and real-time or off-line analysis of data from a single worker or a group of workers. The mobile computing software synchronizes and controls these devices, collecting data from the different sensors, performing real-time analysis to obtain exposure information that is simultaneously mapped against research-based criteria and expressed as risk levels related to, e.g., WMSDs. 

This information can then be used for risk assessment and exposure measurements, in which the system can provide a report of the exposures or risk levels for single or multiple workers performing single or multiple tasks based on recordings of shorter or longer time periods (minutes or several hours), to provide a snapshot or a general picture of the exposure and risk level. Such information can then be applied when prioritizing resources for measures and to support the design of measures such as workstation layout improvements. Additionally, the real-time exposure information from one or multiple workers can be used by ergonomics experts such as occupational health service personnel or professional ergonomists for real-time evaluation of worker training [47,71]. Alternatively, the real-time analyzed data can be fed back to the wearer in two ways. One is to convey the current exposure level (*real-time exposure level)* via concurrent (real-time) feedback. The other way is *accumulated exposure level* (using terminal feedback [72]), which is derived from a longer measurement period, ranging from minutes to hours, depending on the application. Depending on the need, different types of feedback modalities can be provided, such as visual on a screen, auditory through earphones, or tactile via haptic actuators [41]. The intention of feedback directly provided to the worker is to make the worker aware of hazards arising from the individual work technique, as well as poorly designed work environments such as equipment or workstation layout. 

### 1.4. Aim

The aim of this study was to (1) describe the technical aspects of a newly developed haptic feedback module of the Smart Workwear System platform and (2) to evaluate its user experience and usefulness for reducing biomechanical loads in light repetitive manual handling operations by targeting work technique training and workstation design. The usefulness of the system included the system’s efficiency in reducing time spent in adverse upper-arm postures, while also considering the discomfort and user learning. The haptic module tested was designed for research applications, but the study aims to test the usefulness of haptic feedback for applications beyond research.

### 1.5. Article Structure

The rest of this article is organized as follows. Section 2 describes the Smart Workwear System haptic feedback module, and the methodology of the system application and user evaluations of the module. Section 3 presents the results from the evaluation including kinematics postural data, ratings of discomfort/pain and user experience. Section 4 contains a discussion on the potential and limitations of the Smart Workwear System and the haptic feedback module. The findings from the system application and user evaluations are discussed considering effectiveness, clinical relevance, and implications. The system application and user evaluations methodology are discussed considering its strengths, limitations and research opportunities. Finally, the conclusions are presented in Section 5.

## 2. Methods

### 2.1. The Smart Workwear System Haptic Feedback Module

**Figure 2:**
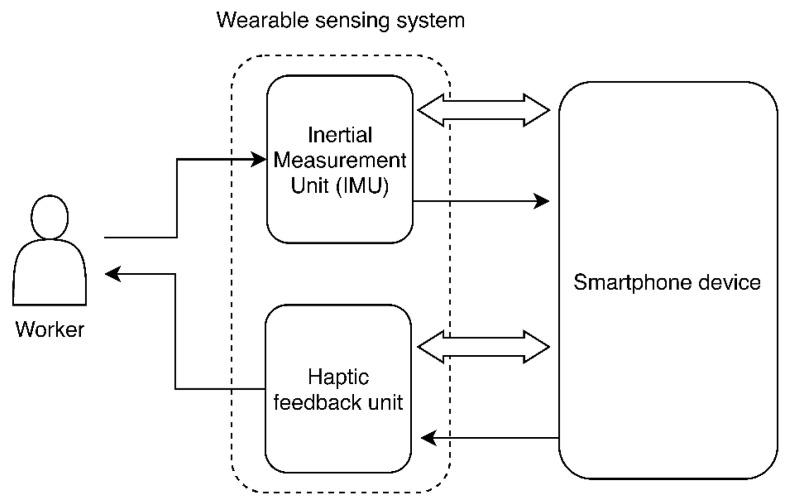
Conceptual depiction of the Smart Workwear System haptic feedback module. Bold black arrows indicate data or actuation flow, while white block arrows illustrate wireless communication flow.

The Smart Workwear System haptic feedback module (Figure 2) is non-invasive and ambulatory, monitors body movements and postures by using IMUs, and provides feedback to the wearer by using a vibrational actuator (haptic feedback unit). The kinematic data from the IMUs are sent to a smartphone device, which stores and automatically evaluates this information with research-based ergonomic criteria [47], using the ‘ErgoRiskLogger’ application. 

Based on this, the wearer is provided with haptic feedback either simultaneously with task execution (concurrent feedback) or after task execution (terminal feedback), using the haptic feedback unit.

**Figure 3:**
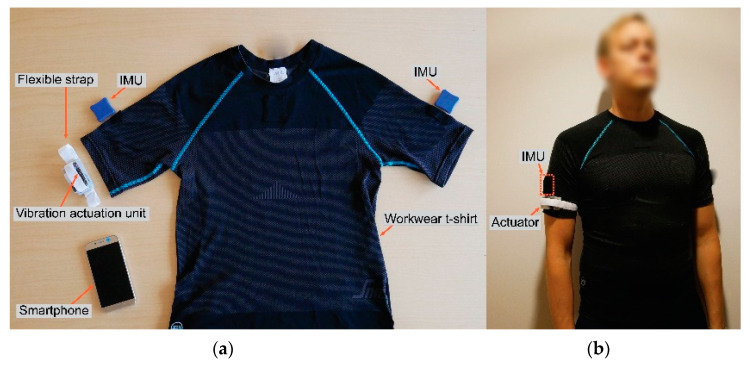
(**a**) The Smart Workwear System, here, (**b**) fitted on the human body with two inertial measurement units (IMUs) for recordings of work postures of the upper arms and to provide haptic feedback on the dominant arm. The hardware components in this module example include a customized workwear T-shirt, two IMUs, one vibration actuation unit, and a semi-elastic textile strap, and one smartphone.

When applied for measurements and feedback on postures and movements of the trunk and upper arms, the IMUs are placed in embedded pockets of a stretchy workwear shirt (9418, LiteWork, Snickers Workwear, Hultafors Group Sverige AB, Bollebygd, Sweden; fabric, 58% polyamide and 42% polyester 37.5^®^; mass, 165 g/m) customized to a short-sleeved shirt (Figure 3). The trunk IMU pocket is placed at the upper back at the level of the thoracic vertebrae 1–2 [73], and the arm IMU pockets are placed bilaterally on the arm sleeves below the insertion of the deltoideus muscle [74,75].

#### 2.1.1. Inertial Measurement Unit

The IMUs (LPMS-B2 IMU, LP Research, Tokyo, Japan; size, 39 × 39 × 8 mm; mass, 12 g) measure acceleration and angular rate in a three-dimensional local reference system. This allows the recording of postural and kinematic data, which are transmitted to the smartphone device via BLE communication standard (Bluetooth version 4.1, Bluetooth Low Energy). The 3-axis accelerometer in the IMUs were configured with a total range of ±4 G and the 3-axis gyroscope with a range of ±500°/s, both recorded with a 16-bit resolution.

**Figure 4:**
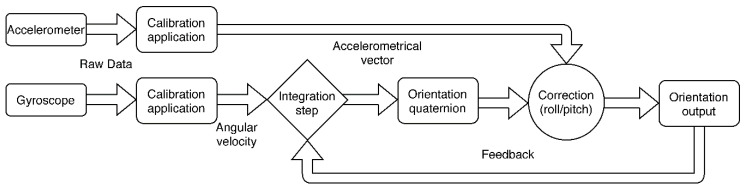
Simplification of the filter. Orientation initially determined by an integration of the angular velocity extracted from the gyroscope after calibration. Orientation is corrected (roll and pitch) with the acceleration vector.

The accelerometer and gyroscope data, acquired with a frequency of 25 Hz, are employed to capture the position of the upper arm with the use of an extensive complementary Kalman filter working as a sensor fusion algorithm. This filter estimates the state of the system and the estimate’s variance, with the state variables defined as the orientation quaternion. Quaternion representation is used in order to avoid angular singularities [76]. The estimate performed by the filter is continuously updated, using the accelerometer and gyroscope measurements from the IMU, as depicted in Figure 4. Initially, estimates of the state variables are produced. The next observed measurement updates the state variables, using a weighted average, where more uncertain measures have a lower weight. This sensor fusion is implemented in the STM32 microcontroller of the LPMS-B2 IMU as part of the IMUcore software modules (LP Research, Tokyo, Japan), allowing users to obtain the quaternion output of the angular position with a frequency of 25 Hz.

After establishing a connection, the quaternion output, the accelerometer, and the gyroscope raw data are continuously transmitted to the smartphone device for real-time haptic feedback performance by the ErgoRiskLogger application, and additionally stored for post-analysis usage.

#### 2.1.2. Haptic Feedback Unit

**Figure 5:**
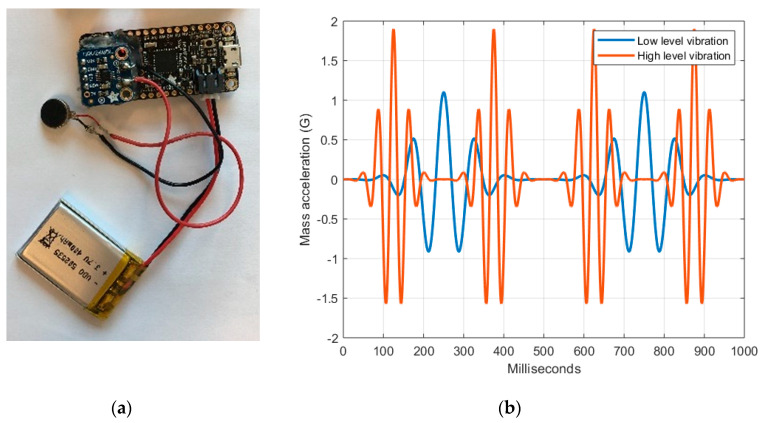
(**a**) Adafruit Feather 32u4 Bluefruit development board connected to an eccentric rotating mass vibration motor and powered by a battery; and (**b**) a depiction of the acceleration of the eccentric rotating mass of the vibrational motor. The first level (in blue) consists of two lower-intensity oscillations, while the second level (in red) consists of four higher-intensity oscillations, for 1000 milliseconds. Users perceived the first level as less intense than the second.

The vibrational actuator (haptic feedback unit) employed was a Ø12 mm eccentric rotating mass vibration motor (Precision Microdrives, London, United Kingdom) (Figure 5a). The actuator was controlled with an Adafruit Feather 32u4 Bluefruit (Adafruit Industries, NY, USA), a compact (51 × 23 × 8 mm) development board with an ATmega32u4 8 MHz processor, equipped with a BLE (Bluetooth version 4.1, Bluetooth Low Energy) module and powered by a lithium-ion polymer 3.7 V 500 mAh battery. The control was executed by a customized control software programmed in C++.

The haptic feedback unit worked as a peripheral node, with no data processing performed by its internal control software. The control software executed the necessary routines to (a) establish a Bluetooth connection with the smartphone device, in order to actuate or report about the status of the sensor and battery, by demand of the software present in smartphone device, and (b) perform the vibration actuation, to convey feedback to the worker. When predetermined Euler upper-arm elevation angle thresholds were exceeded, as detailed in Section 2.2 Test Settings, the control software of the smartphone would request an actuation on the haptic feedback unit. This was made making use of two distinct vibration intensity levels (Figure 5b), produced by the modulation of the pulse duration of the vibration.

#### 2.1.3. Data Collection and Analysis System

**Figure 6:**
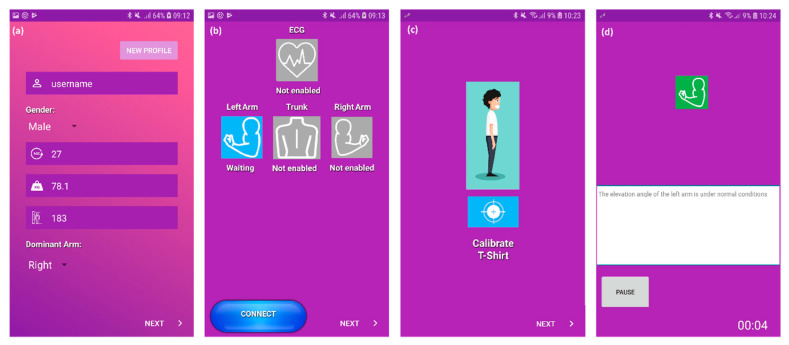
Screenshots of the Android smartphone application ‘ErgoRiskLogger’: (**a**) splash screen to enter wearer identification with a unique identifier and biometrical information (sex, age, weight, height, and selection of the dominant arm); (**b**) user interface screen for the selection of sensors, which is limited to the dominant arm of the user (wearer) for this study; (**c**) IMU and haptic feedback calibration screen, in order to establish a zero elevation position and test vibrational intensity; and (**d**) screen with real-time feedback with additional text report and color-coded icons.

Data collection and real-time exposure calculation and feedback were performed by the ‘ErgoRiskLogger’ software application (Figure 6), which functioned as an edge-computing node in a Samsung Galaxy A7 (Samsung Group, Seoul, Korea). The software was developed for smartphone devices with the operative system Android 7.0 Nougat or higher versions and was accessible and configurable employing a graphical user interface (GUI).

As a first step, the GUI requested an anonymized user identification, followed by the entry of the wearer’s gender, age, weight, and height. Afterwards, the wearer was offered a selection of sensor and actuator devices to connect to, which can be located in different preset positions of the wearable system. Once the connection to the selected sensors was requested by the users, the software initiated a Bluetooth communication standard connection petition to the media access control (MAC) address of each IMU and haptic feedback unit, allowing to univocally connect to each sensor as an independent node. The connection to these sensors made use of a standard class 2 Bluetooth host interface with serial protocol profile support. Connection to the device took approximately 5 s, with a 30 s timeout established to abort the connection attempt and present a warning to the user. Additionally, if a sensor was disconnected during operation, the system would warn the user and try to connect to the sensor node a preset number of times, after which, if failed, it would inform the user that further reconnection to the sensor had to be performed manually. In the same manner, the IMU and haptic feedback unit had an internal routine to stop reconnection attempts to the smartphone after a preset number of times. Once the sensors and actuators were connected, the software initiated a calibration, synchronization, and evaluation procedure. This procedure (a) initialized the referential system of the IMU, by establishing a neutral position of the upper arm as the origin of the reference system and (b) tested the vibrational perception from the wearer, by performing both vibration intensities sequentially in order to verify that device was properly located and that feedback could be perceived.

Upon request, the software application started recording the raw data and angular orientation extracted from the IMU, which is expressed in quaternions, referenced to the initialized referential system. These referential units are transformed into Euler angles, which are used by both the system operator (e.g., the ergonomist, see Figure 1) and the real-time risk assessment algorithm. Additionally, the timestamps in the application of haptic feedback are also stored.

The start of the recording of the sensor data triggers the commencement of the real-time risk assessment. Additionally, the GUI displays color-coded icons and a textual report, which inform the ergonomist of the risk level. Additionally, this textual report is stored, associated with the evolution of exposure over time.

All raw sensor data are synchronized with the addition of timestamps and stored for post-analysis in the smartphone device, individually for each wearer with their unique identification.

### 2.2. Test Settings

#### 2.2.1. Participants

Sixteen participants (nine women and seven men), were recruited from the student and staff population of the university where the study was performed. Twelve reported right-hand dominance, and four reported left-hand dominance. Their mean (SD) age was 25 (8) years, body mass 70 (13) kg, stature 170 (13) cm, elbow height 105 (8) cm, and elbow–fingertip length 44 (4) cm. All participants had ≤3 months’ experience of mail sorting and were thus considered novices. Only participants without musculoskeletal discomfort or disorders that could hinder the mail sorting were included. All participants gave written consent prior to participation, and the study was approved by the regional ethical committee in Stockholm, Sweden (2017/1586-31/4).

#### 2.2.2. Equipment and Feedback Thresholds

The participants were equipped with the customized Smart Workwear T-shirt, and two IMUs were placed bilaterally in embedded upper-arm pockets where the upper edge of the IMU was approximately positioned below the insertion of the deltoideus muscle [74]. The haptic feedback unit with the integrated Bluetooth transmitter was positioned approximately 5 cm distal of the IMU on the participants’ dominant arm and secured by using a semi-elastic textile strap (Figure 3b). The reference position for the upper arm (to denote 0° upper-arm elevation) was recorded with the subject standing upright, looking forward at eye-level, and with relaxed arms hanging down and with the palms facing towards the body [38,69]. Only postural data from the dominant arm were used for the haptic feedback as well as for evaluating the effect of the haptic feedback training. 

The concurrent haptic feedback (Figure 5b) was provided for upper-arm elevation angles of ≥30° and ≥60° relative to the upper-arm reference position, with the aim of augmenting the awareness of these upper-arm elevation threshold angles. These two elevation angles are correspondingly assigned to the two vibrational levels, ordered in intensity level. The rationale for using these thresholds was that upper-arm elevation angles exceeding 30° for >50% of the work time and 60° for >10% of the work time [77], and upper-arm elevation angles exceeding 45° for >15% of the worktime [4] have been associated with increased risk of MSDs in the neck–shoulder region.

#### 2.2.3. Procedure

**Figure 7:**
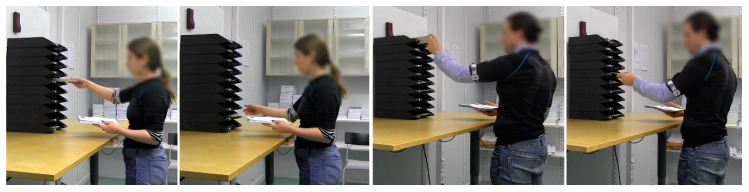
Example of postures of two participants during the mail sorting.

The haptic feedback module was tested on simulated mail sorting, since postal delivery workers have reported a high prevalence of upper-extremity MSDs compared to the general working population, and mail sorting has been reported as one of their most physically demanding work tasks [78], involving frequent movements of the upper arms. The simulated mail sorting task was performed in a laboratory setting, in which a mail sorting workstation was designed comprising a height-adjustable table and a letter tray stack (Figure 7).

The task comprised the sorting of 30 randomly ordered letters (marked 0–9) in their corresponding letter tray (marked 0–9) under two experimental conditions: (A) an intervention condition with predetermined positions of the letter tray stack and where the participants received verbal ergonomics instructions to reduce adverse postures (Ergo-Instr. 1 and 2) or verbal instructions in combination with haptic feedback (Haptic-FB 1 and 2), and (B) a workstation design condition (WS-design) where the subjects were instructed to arrange the position of the ten letter trays to minimize their upper-arm elevation receiving verbal ergonomics instructions (WS-Design 1 and 2) or verbal ergonomics instructions in combination with haptic feedback (WS-Design 3). Each participant performed a practice session before the study to become familiar with the mail sorting task, subjective ratings of perceived discomfort/pain, using the Borg CR10 scale [79] and a body map [80], and the *general work instruction* that was based on previous psychophysical studies to determine acceptable workloads [81]. No time restrictions were given; instead, the subjects were instructed to imagine the mail sorting task as being part of their normal job and performing the task at a pace which they could sustain for two hours per workday for five days per week, and that the task was not a competition (i.e., the *general work instruction*).

**Figure 8:**
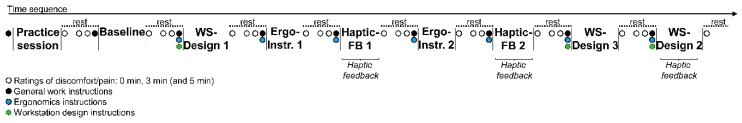
Time sequence of the test comprising the practice session, the baseline, the intervention condition (Ergo-Instr. 1 and 2, and Haptic-FB 1 and 2), and the workstation redesign condition (WS-Design 1–3).

After the practice session, the participants performed a baseline session followed by seven sessions (Figure 8), in all of which they sorted 30 randomly ordered letters, using their dominant upper arm. Before the baseline session, the *general work instruction* was repeated, and for the seven following sessions an ‘ergonomics instruction’ (i.e., Ergo-Instr.) was provided in addition to the *general work instruction.* For the ‘ergonomics instruction’, the participants were instructed to reduce the upper-arm elevations by keeping the elbow close to the trunk when performing the mail sorting. For three of the sessions, the participants received concurrent haptic feedback. Before these three sessions, the participants were additionally instructed that the system would give them vibration feedback on their dominant upper arm if they did not keep their elbow close to their bodies. For the workstation design sessions, the participants were additionally instructed to arrange the position of the letter trays to minimize their upper-arm elevation.

Immediately after sorting the last letter of each session, the participants rated their perceived discomfort/pain, using the Borg CR10 scale and the body map to indicate the discomfort/pain intensity level and the locations. A minimum rest period of 3 min was provided between each session to avoid the accumulation of fatigue. After the 3 min rest period, the participants repeated the discomfort/pain ratings. In the event that they reported a CR10 discomfort/pain intensity level of “1” (i.e., “very weak” discomfort/pain) or more, an additional rest period of 2 min was provided [82].

For the baseline and the intervention sessions, the letter tray stack was placed on a height-adjustable table with the tabletop vertically positioned at each participant´s elbow height (wearing shoes). The front of the stack was positioned at a horizontal distance equivalent to each participant´s elbow–fingertip length, defined as the horizontal distance from the back of the elbow to the middle fingertip [83]. For the three workstation design sessions, the height-adjustable table was lowered to a position with the tabletop positioned between the participant’s knee and waist level and the letter tray placed on a separate table. Thereafter the participants were instructed to freely arrange the workstation (letter tray and height-adjustable table) to minimize demanding work postures. Thereafter, the participants sorted the 30 letters in this self-designed workstation. All sessions were videotaped.

After having finished the experimental conditions, a semi-structured interview was conducted and recorded for each participant. The questions focused on the participants’ experiences of discomfort/pain, the workwear system and its usage, the task, workplace arrangements and learnings from the sessions (see Appendix A). The recorded interviews were analyzed by extracting meaningful entities from each participant and question [84].

### 2.3. Statistical Analysis

To statistically test the effect of the vibrotactile feedback and the verbal instruction on the upper-arm elevation angle compared to the baseline, a within-subject analysis with pairwise comparisons was employed. The intervention effect was tested by comparing the proportion of the accumulated time in dominant upper-arm elevations ≥30°, ≥45°, and ≥60°, and the arm angles (50th, 90th, 95th, and 99th percentiles) at baseline compared with the intervention scenarios. The data were checked for symmetric data distributions, and the Wilcoxon signed-rank test was applied to statistically test the difference in exposure between the scenarios. A *p*-value of <0.01 was used to denote statistically significant differences, and statistical analyses were performed in IBM SPSS Statistics 26 (Armonk, NY, USA).

## 3. Results from System Application and User Evaluations

### 3.1. Effect of Haptic Feedback and Verbal Ergonomics Instructions on Upper-Arm Posture

**Figure 9:**
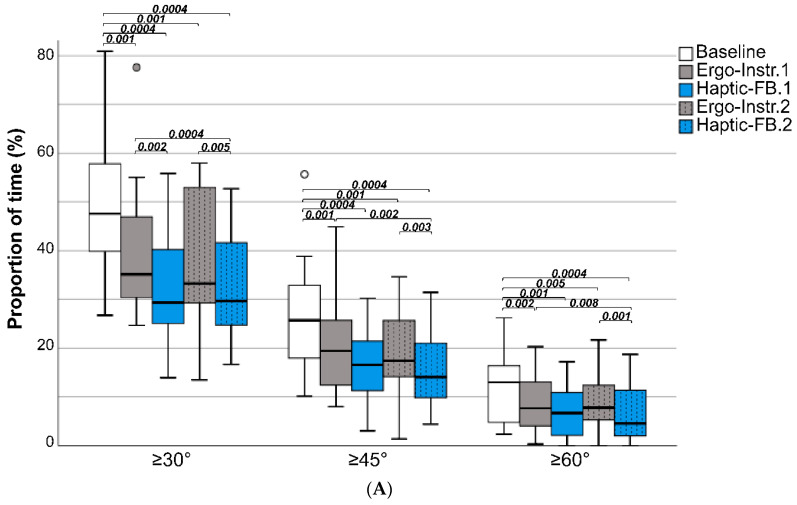

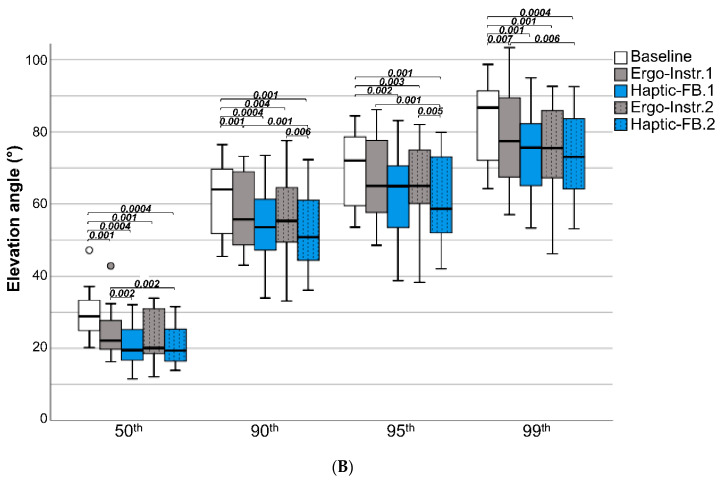
(**A**) Accumulated time of the dominant upper-arm elevations ≥30°, ≥45°, and ≥60°, and (**B**) the 50th, 90th, 95th, and 99th percentile dominant upper-arm elevation angles of the baseline and the intervention scenarios where the participants received verbal ergonomics instructions (Ergo-Instr.) or verbal ergonomics instructions in combination with haptic feedback (Haptic-FB). The boxplots show the median, the interquartile range, and the max and min values. Statistically significant (*p* < 0.01, Wilcoxon signed-rank test) differences between the scenarios are displayed with their corresponding *p*-values.

As shown by the descriptive data (Table 1 and Figure 9a), significantly less accumulated time was spent in upper-arm elevations ≥30°, ≥45°, and ≥60° when given verbal instructions (Ergo-Instr. 1 and 2 scenarios), and when additionally given haptic feedback (Haptic-FB 1 and Haptic-FB 2) when compared to the baseline. Compared to the baseline, the group median accumulated time declined by 24–65% (Table 1). The same trend was also found when examining the 50th, 90th, 95th, and 99th percentile upper-arm elevation angle (Figure 9b), where a significant decline in elevation angle was observed for all scenarios (except for the 95th percentile angle for the Ergo-Instr. 1 scenario) when compared to the baseline, where the group median angle decreased by 10–33%. Further, no significant difference in accumulated time or angle (P50–99) was observed when comparing the Ergo-Instr. 1 to the Ergo-Instr. 2 scenarios, and when comparing the Haptic-FB 1 to the Haptic-FB 2 scenario However, when comparing Ergo-Instr. 1 and Ergo-Instr. 2 scenarios with the Haptic-FB 2 scenario, significant decline was observed in the accumulated time in upper-arm elevations ≥30°, ≥45°, and ≥60°.

### 3.2. Workstation Design Effect

**Figure 10:**
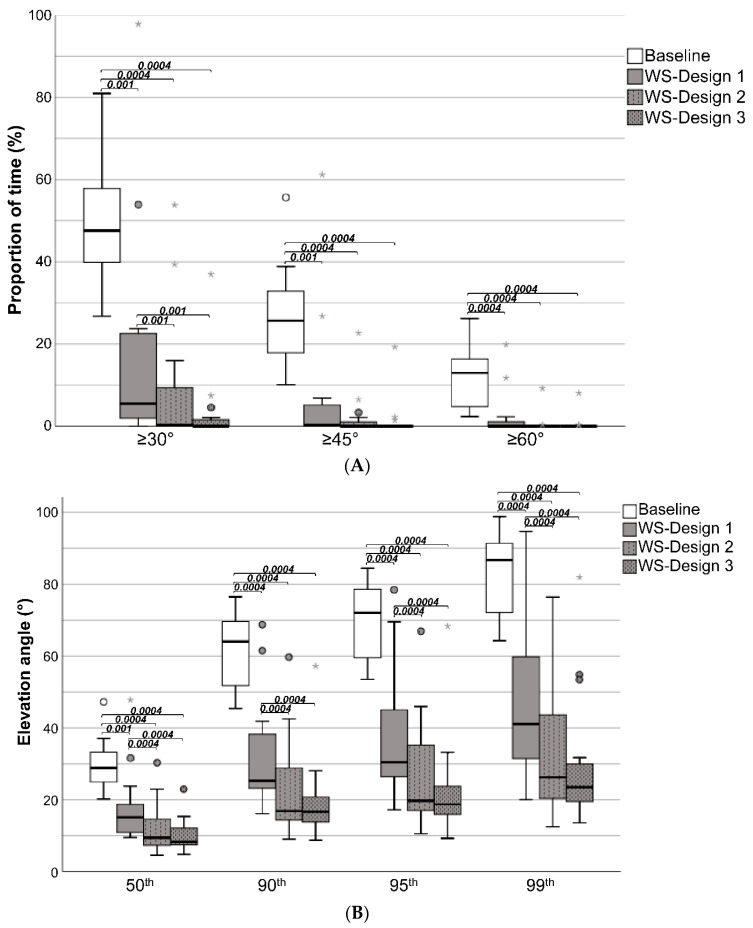
(**A**) Accumulated time of the dominant upper-arm elevations ≥30°, ≥45°, and ≥60°, and (**B**) the 50th, 90th, 95th, and 99th percentiles of dominant upper-arm elevation angle in the baseline and the workstation design (WS-Design) scenario. The boxplots show the median, the interquartile range, max and min values. Statistically significant (*p* < 0.01, Wilcoxon signed-rank test) differences between the scenarios are displayed with their corresponding *p*-values.

As shown by the descriptive data (Table 2 and Figure 10a), significantly less accumulated time was spent in upper-arm elevations ≥30°, ≥45°, and ≥60° when given the possibility to redesign the workstation (WS-Design sessions) when compared to the baseline. Compared to the baseline, the group median accumulated time declined by 88–100% (Table 2). The same trend was also found when examining the 50th, 90th, 95th, and 99th percentile upper-arm elevation angle (Figure 10b), where a significant decline in elevation angle was observed for all scenarios when compared to the baseline, where the group median angle decreased by 48–74%. Further, when having received haptic feedback training, a significant decrease of the accumulated time in upper-arm elevation angle ≥30° was observed with a decrease of 94% (not shown in the table). The decline after having received haptic feedback was also observed in the angle (P50–99), where a decline of 33–37% was observed (not shown in the table).

### 3.3. Ratings of Discomfort/Pain

**Figure 11:**
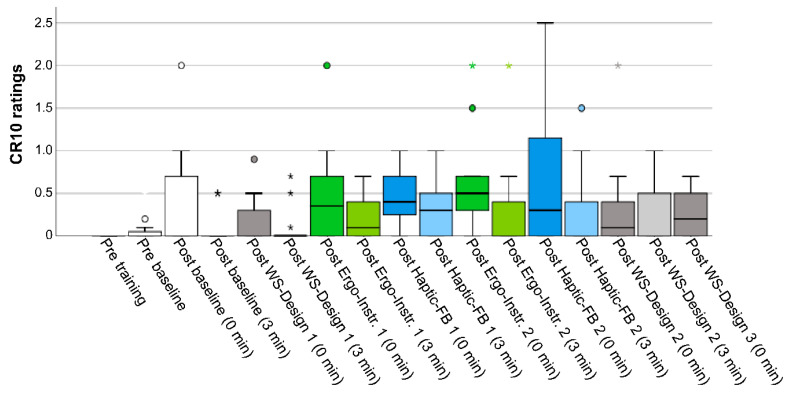
Borg CR10 discomfort/pain ratings before the training and baseline sessions, and after finishing each session (0 and 3 min after end). The values include the peak discomfort/pain ratings for each participant for each measurement occasion. The boxplots show the median, the interquartile range, and the max and min values.

As shown by the descriptive data (Figure 11), low CR10-ratings of discomfort/pain were reported for all sessions. The mean CR10-ratings for all sessions immediately after finishing the task were 0.41 (SD 0.28), and 0.21 (SD 0.35) after 3 minutes’ rest, where a value of 0.5 corresponds to an *extremely weak (just noticeable) discomfort/pain*. The mean CR10-ratings were <0.7 for each session end (0 min), and <0.5 after 3 minutes’ rest. A few participants rated the discomfort/pain as weak/light (2.0) or slightly more (2.5) following the end of the sessions. These few ratings were generally reduced to around 1.0 (*very weak discomfort/pain*) after a rest period of 3 min. Twelve of the participants did not at any point state their discomfort/pain to be more than *very weak*, while four participants reported their discomfort/pain to be more than *very weak* at least once during the entire trial, and located in the following body regions: right/left shoulder (N = 2), right/left elbow (N = 1), and upper/lower back (N = 2). None of the participants, however, reported discomfort/pain to be more than *very weak* immediately following the last test session (WS-Design 3). It should be noted that, when asked to rate the perceived *discomfort/pain*, the participants tended to base their discomfort/pain-ratings on perceived discomfort rather than perceived pain.

### 3.4. User Experiences

The task was in general considered monotonous, and 14 participants in the interviews reported having felt some kind of discomfort during the sorting. The discomfort felt during the task made most participants aware of the problems of the vertical work height and the horizontal reach distance. A few participants spontaneously tried to change their work techniques to make sorting more comfortable. The haptic feedback made all but one of the participants more aware of their work technique. The most frequent response was that the feedback reminded them of their posture all the time. However, four persons mentioned that the feedback made them more distracted or stressed at the beginning, even if this feeling tended to disappear fairly rapidly. The feedback was also the most important input for redesigning the workstation, even though the participants also used their experiences from body discomfort in addition to the haptic feedback as input to the redesign. However, the feedback was considered more powerful by most participants. The participants rearranged and designed their workstation to get a lower vertical height of the racks (between waist and elbow height), closer to the body, angled in relation to the reaching direction, and with an adjusted work surface height. Fifteen participants considered the haptic feedback positively and one was indifferent. The participants used words such as fun, exciting, interesting, but also unusual. Some of them also pointed to the trustworthiness of the feedback from the system.

## 4. Discussion

### 4.1. The Smart Workwear System

This paper introduces a new module-based ambulatory system: the Smart Workwear System, targeting work-related physical and psychosocial stressors that can have adverse or promoting effects on health and direct or indirect effects on work performance.

It supports risk assessment, work technique training, and work and workplace design, at the same time as it reduces the need for the involvement of skilled trainers and ergonomists. Moreover, substituting observations with measurements has the potential to increase accuracy and reproducibility of the measurements. Using a modular platform, a range of sensor types can be connected depending on the specific need of each situation. To date, sensors for the recording of work posture and movements, heart rate, respiratory rates, energy expenditure, and force exertions have been tested in real work environment contexts [44,47,70,71]. As demand rises for the assessment of an increasing number of risk factors, the Smart Workwear System is continuously being developed to include additional risk factors addressed in the research literature, allowing for more sensors and with more analysis software. There are many wearable solutions that have an origin in sports, rehabilitation and fitness applications and of different technological sophistication (see, e.g., www.hexoskin.com and www.swedishposture.com). Many of these solutions are capable of recording various signals representing posture, load, or other physiological measurements, but they are typically designed for just one or a small number of specific types of analyses and targeting a few specific problems/issues. In contrast, the Smart Workwear System constitutes a platform allowing sensors, microprocessor devices, mobile/stationary computers, and servers to be integrated into a complete solution as a supportive tool for workplace-related ergonomic tasks such as risk assessment, workstation design, etc. The software implements algorithms to assess the risks of musculoskeletal disorders and injuries, and it implements methods for work technique training that are based on contemporary research. Every application of the Smart Workwear System includes only the components that are required for the task at hand, but it can be expanded at any time with additional components should the need arise.

The idea behind the risk assessment function is that work tasks and workplaces should be assessed, not individuals. Therefore, it is desirable that several workers perform the same tasks at the same workplace so that the influence of individual differences can be limited. From an ethical and an integrity point of view, the ownership of individual data is of importance. Previous research indicates that employees are positive about receiving individual feedback on work-related exposures, but they like to retain ownership of the collected data, and the mandate to decide with whom the data are shared [85]. This highlights the importance of possibilities for the anonymization of the collected data at all steps of the data management, including storage. 

### 4.2. The Smart Workwear System Haptic Feedback Module

The haptic feedback module was developed for automatic real-time work technique training for both newly employed and currently employed staff working in physically demanding jobs. The chosen feedback modality (i.e., concurrent feedback) is intended for short training periods of about 10–30 min. For longer training periods, other feedback modalities should be considered, such as terminal feedback where the feedback is provided after task execution [72]. While this application uses haptic feedback as a strategy to mimic and augment intrinsic signals of discomfort or pain, other feedback modalities might be suitable, such as visual or auditory feedback [41]. However, the use of visual feedback may be less suitable when the task requires continuous visual control, and auditory feedback may be unsuitable in noisy work environments.

Some limitations of the system have been identified. Similar to several other systems that record human movements and postures [45,46], the haptic feedback system is sensitive to the initial IMU and haptic feedback calibration, for which the system currently has no automatic verification routine to validate if the angles and positions that are established as a reference are coherent and within a certain tolerance in a global reference system. Currently, the calibration has to be performed when the IMUs are mounted on the wearer. Recent validation of the system [86] with forty participants shows excellent accuracy and precision of the system when validated reference positions are used for calibration of the global postural angles.

Additionally, strenuous movement may shift the location of the IMU, causing motion artifacts that in some situations can be transformed into false positive feedback to the worker or shift the location of the haptic feedback unit, not conveying feedback correctly to the worker if the contact surface of the skin with the vibration actuator is reduced. To limit potential motion artifacts, a filtering algorithm was applied to the angular acquisition. This translated into a small delay (0.5 s) in the feedback received by the worker, for all motions.

Concerning the angular measurement by the IMU, this was limited to the use of the three-axis accelerometer and gyroscope. Small errors introduced from the gyroscope measurements in the integration step have an exponential influence on the angular calculation result. Orientation data from the gyroscope, which was corrected with information from the accelerometer (roll and pitch angles), should be further improved with corrections from the magnetometer (yaw angle), to calculate orientation information with higher accuracy and robustness against drift over time. However, as previously shown by Robert-Lachaine et al. [87], the use of magnetometers may not be suitable in work situations with significant local magnetic fields. 

The system was designed to operate in a personal area network, making use of Bluetooth Low Energy for connection and data transmission. This limits the distance between the data receiver (a smartphone device) and the operator wearing the wearable system. If this distance restriction is exceeded, disconnection ensues, which could cause the loss of data for a certain time. Currently, the system executes a warning to the wearer and a reconnection routine with the IMU and haptic feedback system, but future improvements can introduce local storage in the sensors for a limited period, mitigating the risk of data loss. Additionally, even though battery management routines are implemented, both in the software of the sensors and the ErgoRiskLogger software application, and devices that support and use Bluetooth Low Energy transmission have been selected, the battery life of the complete haptic system is currently lower than a complete 8 h workday, this depending on the need to apply haptic feedback more frequently for a certain wearer or based on the active interaction of the user with the smartphone device. Future implementations should improve the efficiency of the data packages transmitted and investigate other vibration implementations which can convey similar feedback levels with lower energy consumption. The current technical version of the haptic module is currently limited to research. For use beyond research applications, the technology needs to be converted into a more robust solution and tested regarding its durability and long-term service life.

### 4.3. System Application and User Evaluations

#### 4.3.1. System Application and User Evaluations—Major Findings, Clinical Relevance, and Implications

The system application and user evaluations present the first evaluation of this specific haptic module of the Smart Workwear System, using a single haptic feedback unit. The study found a reduction of the accumulated time in upper-arm elevation following both the verbal ‘ergonomic’ instructions and the haptic feedback. While both strategies were effective, the haptic feedback in combination with verbal instructions had significantly larger effects on reducing the accumulated time in upper-arm elevation when compared to using verbal instructions only.

A substantially reduced proportion of accumulated time was observed among the participants when provided solely with verbal ‘ergonomic’ instructions (24–41%) and reduced further when provided with haptic feedback training in combination with verbal ‘ergonomic’ instructions (36–65%). This reduction in accumulated time in upper-arm elevation related to the haptic feedback is larger than has been observed previously, where reductions in accumulated time in upper-arm elevation angles of 4–32% were observed [69]. These differences might reflect the proportional postural load attributed to the individual work technique, hence the potential that can be targeted by using the haptic feedback training for work technique improvements. Clearly, all adverse exposures cannot be reduced solely by work technique improvements and they are also to a large extent influenced by, e.g., the work/workstation design. Given the results from the interviews, the haptic feedback module had the potential to contribute to improvements in work/workstation design. 

If the exposures during mail sorting at baseline are extrapolated to 8 h of work, the average accumulated time in upper-arm elevations ≥30°, ≥45°, and ≥60° relative to the reference would be approximately 228, 123, and 62 min, respectively. Such exposures exceed the action level proposed by Arvidsson et al. [77], which stipulates that the upper-arm elevation angle should not exceed 60° for more than 10% of the worktime (i.e., 48 min). Additionally, the proportion of time ≥45° exceeds 15% of the worktime (i.e., 72 min), i.e., a level associated with increased risk of MSDs in the shoulder region [4]. Following the haptic feedback (Haptic-FB 2) and extrapolated to 8 h work, the mean time for elevation angles ≥45° would drop to 67 min, and the mean time for elevation angles ≥45° to 22 min, both below the levels associated with MSDs in the shoulder region [4,77]. Hence, this indicates that clinically relevant reductions in exposure were obtained following the haptic feedback. 

#### 4.3.2. System Application and User Evaluations—Methodological Considerations and Limitations

Previous studies from industrial type manual handling operations have shown significantly longer task execution times when receiving direct feedback training [45,69], which may be attributed to actions taken to alter the work technique. Given that increased task execution time reduces productivity, the increased execution time can have negative effects on work performance. For the present study, however, no statistically significant difference in the time to complete the task was observed for either the verbal instruction sessions or the haptic feedback sessions when compared to the baseline, although a slight tendency to longer execution times was observed for the haptic feedback sessions.

It is possible that the relatively low complexity level of the simulated mail-sorting task contributed to this, as only minor, relatively obvious, work technique changes were needed in order to reduce the occurrence of the haptic feedback signal (and load levels). For work involving operations with a higher level of complexity, the situation may be different.

Additionally, the total feedback training period in the current study and in Lind et al. [69] was less than 15 min, so the effect on reduced productivity is limited and should rather be compared with the time needed when using other training programs. Interestingly, no significant difference was observed in the accumulated time of upper-arm elevation when comparing the first and second haptic feedback sessions (Figure 9a), or when comparing the first and second verbal instruction sessions. This indicates that the participants rapidly implemented strategies that reduced the time in the adverse arm postures. These findings may have practical implications for how long a time the feedback is needed for improving the work technique, and indicates that the haptic feedback training is time-efficient.

Feelings of discomfort during the performance of the task were an obvious source of (intrinsic) feedback and identified problems with the workstation design. Stronger discomfort seemed to be a stronger motivator to change work technique and the workstation design. However, it seemed as if the haptic feedback was a stronger input channel, especially for those who felt no or weak discomfort. In that situation, the haptic feedback strengthened or even replaced the intrinsic body signals. One potential risk with this work technique training application is that the training decreases the load on the intended body part, but instead increases the load on another body part. As long as this does not cause adverse loads on the other body parts, the technique change can be considered desirable. If discomfort arises in other body parts, however, this must be considered and addressed. This aspect is of course an important issue to be aware of when applying all types of work technique training. Based on visually observing the participants, a changed work technique was observed, including having the hands closer to the body and, for some, using wrist flexion. However, no clearly increased musculoskeletal discomfort in those regions was reported, but given the short time span, such effects may arise after longer time periods. Therefore, monitoring of the long-term effect following work technique changes is desirable.

The feedback modality used (concurrent haptic feedback) is based on error augmentation/amplification aiming at raising the awareness of hazardous work situations, and thereby promoting alternative work techniques strategies that are more beneficial from an MSD-prevention perspective. Alternatively, augmentation of sound work techniques strategies could have been used. As previous studies have concluded, the use of both concurrent feedback and error augmentation may be less efficient when feedback is provided for longer time periods and may increase the risk of inhibiting the wearer from responding to cues provided by the body´s intrinsic feedback system [72]. This might also result in extrinsic feedback dependency [88,89,90]. Therefore, using the concurrent haptic investigated in the present study may be less advisable if the feedback training is to be used for several hours or workdays.

The case study included only novice participants and a manual sorting task with a relatively low complexity level, so generalization to experienced workers, as well as tasks with a higher complexity level, should be made with caution. While the use of novice participants may be less suitable for some investigations, this can be justified when examining effects in populations of newly hired staff that are inexperienced in the work operations. However, the results from previous studies indicate that the Smart Workwear System can improve the work technique also in experienced workers [69].

To test the potential effect of verbal instructions and haptic feedback, a within-subjects design was applied where each subject´s postural load was compared between all scenarios. To minimize potential effects related to the task order, random assignments of the test scenarios could have been used. However, a randomly assigned order was not seen as an appropriate choice since this would most likely have introduced unwanted spillover effects, where learnings following the haptic feedback could have influenced the work technique during sessions without haptic feedback, as seen from the retained effect following biofeedback training [62,91]. Instead, the subjects were provided with a training period to familiarize themselves with the mail sorting and to ensure that they were sufficiently trained before the baseline. Given the low complexity of the task and to avoid fatigue, a short test trial was chosen. Two researchers were present during the trials and observed for cues indicating the potential need to extend the training period.

To what extent the reduced exposures following the haptic feedback and the verbal instructions are retained, and whether the additional effect of using haptic feedback compared to solely verbal instructions is retained, amplified, or attenuated cannot be answered by this study. A clear advantage of only using verbal instructions is that they can be easy to administer, at least if trained instructors are available. Experience from previous studies on assembly workers [47] indicates, however, that the workers often tend to forget such verbal instructions when performing cognitively demanding tasks, while constant reminders in the form of concurrent feedback retain the focus on reducing adverse posture despite the high cognitive demands. Although more studies are needed to evaluate the retained effects, some studies indicate promising retained effects following biofeedback training [62,91]. More research is also needed to establish a suitable training program, including guidelines for how long a time the feedback is provided and how often, e.g., once per month or year, etc. Such studies should also consider the maximum suitable number of haptic devices and how this influences the possibility of conveying meaningful information that will facilitate improvements in the work technique.

## 5. Conclusions

The Smart Workwear System is an ambulatory and flexible sensor-based technical solution targeting work-related physical and psychosocial stressors that can have adverse or promoting effects on health and direct or indirect effects on work performance. The haptic feedback module of the system allows work technique training in simulated mail sorting. The module consisted of a customized T-shirt, an IMU, a haptic feedback unit, a smartphone, and an Android-based application to collect, analyze, and send sensor data, using Bluetooth connections. This study shows that the use of haptic feedback for work technique training has the potential to significantly reduce the time in adverse upper-arm postures after short periods of training. The system can increase the awareness of the situations that create adverse upper-arm postures. The haptic feedback was experienced positive and usable by the participants and this kind of extrinsic biofeedback was effective in supporting learning how to improve postures and movements. The feedback also made the participants aware of several different improvements of workstation design, and supported them to design and rearrange the workstation. The system improved the work technique and supported work and workplace design at a relatively low cost with good accuracy compared to alternative methods. The system is flexible and modular, and can thus be adapted to a range of work-related exposures, and sectors of working life and in research. It is concluded that this type of sensorized system, using haptic feedback training, is promising for the future, especially when organizations are introducing newly employed staff, when teaching ergonomics to employees in physically demanding jobs, and when performing ergonomics interventions. Future studies are needed to improve the efficiency of the feedback regimes and to identify limits of how much and what type of feedback information can be beneficial. For use beyond research applications, the technology needs to be converted into a more robust solution and tested regarding its durability and long-term service life.

## Figures and Tables

**Table 1:** The median (M) values in accumulated time in dominant upper-arm elevations ≥30°, ≥45°, and ≥60°, and the 50th, 90th, 95th, and 99th percentile dominant upper-arm elevation angles of the baseline and the intervention scenarios where the participants received verbal ergonomics instructions (Ergo-Instr.) or verbal ergonomics instructions in combination with haptic feedback (Haptic-FB). The proportional differences (diff) of the values obtained in the intervention scenarios when compared to the baseline value, where significant differences (*p* < 0.01, Wilcoxon signed-rank test) are displayed in bold.

Baseline		Ergo-Instr. 1			Haptic-FB 1			Ergo-Instr. 2			Haptic-FB 2		
	M	M	diff (%)	*p*	M	diff (%)	*p*	M	diff (%)	*p*	M	diff (%)	*p*
**Arm elevation (s)**													
≥30°	48.6	35.1	26	**0.001**	29.3	38	**<0.001**	33.2	30	**0.001**	29.7	38	**<0.001**
≥45°	25.7	19.4	24	**0.001**	16.5	36	**<0.001**	17.4	32	**0.001**	14.0	45	**<0.001**
≥60°	13.0	7.7	41	**0.002**	6.7	49	**0.001**	7.8	40	**0.005**	4.5	65	**<0.001**
**Arm elevation (** **°)**													
50th	28.9	22.1	23	**0.001**	19.5	32	**<0.001**	20.1	31	**0.001**	19.4	33	**<0.001**
90th	64.0	55.7	13	**0.010**	53.5	16	**<0.001**	55.3	14	**0.004**	50.9	21	**0.001**
95th	72.0	65.1	10	0.020	65.0	10	**0.002**	65.1	10	**0.003**	58.7	19	**0.001**
99th	86.7	77.5	11	**0.007**	75.7	13	**0.001**	75.5	13	**0.001**	73.1	16	**<0.001**

**Table 2:** The median (M) values in accumulated time in dominant upper-arm elevations ≥30°, ≥45°, and ≥60°, and the 50th, 90th, 95th, and 99th percentile dominant upper-arm elevation angles of the baseline and workstation design (WS-Design) scenario. The proportional differences (diff) of the values obtained in the workstation design sessions 1–3 when compared to the baseline value, where significant differences (*p* < 0.01, Wilcoxon signed-rank test) are displayed in bold.

Baseline		WS-Design 1			WS-Design 2			WS-Design 3		
	M	M	diff (%)	*p*	M	diff (%)	*p*	M	diff (%)	*p*
**Arm elevation (s)**										
≥30°	47.6	5.5	88	**0.001**	0.3	99	**<0.001**	0.0	100	**<0.001**
≥45°	25.7	0.3	99	**0.001**	0.0	100	**<0.001**	0.0	100	**<0.001**
≥60°	13.0	0.0	100	**<0.001**	0.0	100	**<0.001**	0.0	100	**<0.001**
**Arm elevation (** **°)**										
50th	28.9	15.1	48	**0.001**	9.5	67	**<0.001**	8.2	72	**<0.001**
90th	64.0	25.3	61	**<0.001**	16.9	74	**<0.001**	16.7	74	**<0.001**
95th	72.0	30.4	58	**<0.001**	19.7	73	**<0.001**	18.7	74	**<0.001**
99th	86.7	41.1	53	**<0.001**	26.2	70	**<0.001**	23.5	73	**<0.001**

## Appendix A

The following are the questions used in the semi-structured interview:

- How did you perceive the mail-sorting task?
- Did you feel any discomfort during the session?
- How did the discomfort influence you and your way of working?
- How did you perceive the vibration feedback?
- What did you learn from the feedback?
- How did the vibration feedback affect you?
- How did the vibration feedback affect your way of working?
- What made you rearrange the workplace the way you did?
- Is there anything else you would like to add that we did not ask about?

## References

[1] Tompa E., Mofidi A., van den Heuvel S., van Bree T., Michaelsen F., Jung Y., Porsch L., van Emmerik M. (2019). The Value of Occupational Safety and Health and the Societal Costs of Work-Related Injuries and Diseases.

[2] ILO Global Trends on Occupational Accidents and Diseases. https://www.ilo.org/legacy/english/osh/en/story_content/external_files/fs_st_1-ILO_5_en.pdf.

[3] Lötters F., Burdorf A., Kuiper J., Miedema H. (2003). Model for the work-relatedness of low-back pain. Scand. J. Work Environ. Health.

[4] Van Rijn R.M., Huisstede B.M., Koes B.W., Burdorf A. (2010). Associations between work-related factors and specific disorders of the shoulder—A systematic review of the literature. Scand. J. Work Environ. Health.

[5] Sluiter J.K., Rest K.M., Frings-Dresen M.H.W. (2001). Criteria document for evaluating the work-relatedness of upper-extremity musculoskeletal disorders. Scand. J. Work Environ. Health.

[6] NRC (2001). Musculoskeletal Disorders and the Workplace: Low Back and Upper Extremities.

[7] Punnett L. (2014). Musculoskeletal disorders and occupational exposures: How should we judge the evidence concerning the causal association?. Scand. J. Public Health.

[8] Eurofound (2016). Sixth European Working Conditions Survey.

[9] Eurofound (2012). Fifth European Working Conditions Survey.

[10] Marchet G., Melacini M., Perotti S. (2015). Investigating order picking system adoption: A case-study-based approach. Int. J. Logist. Res. Appl..

[11] Baker P., Halim Z. (2007). An exploration of warehouse automation implementations: Cost, service and flexibility issues. Supply Chain Manag..

[12] Beier G., Ullrich A., Niehoff S., Reißig M., Habich M. (2020). Industry 4.0: How it is defined from a sociotechnical perspective and how much sustainability it includes—A literature review. J. Clean. Prod..

[13] EU (1990). Council Directive 90/269/EEC of 29 May 1990 on the Minimum Health and Safety Requirements for the Manual Handling of Loads Where there is a Risk Particularly of Back Injury to Workers.

[14] Eliasson K., Lind C.M., Nyman T. (2019). Factors influencing ergonomists’ use of observation-based risk-assessment tools. Work.

[15] Ivarsson A., Eek F. (2016). The relationship between physical workload and quality within line-based assembly. Ergonomics.

[16] Eklund J. (1997). Ergonomics, quality and continuous improvement—Conceptual and empirical relationships in an industrial context. Ergonomics.

[17] Falck A.C., Örtengren R., Högberg D. (2010). The impact of poor assembly ergonomics on product quality: A cost-benefit analysis in car manufacturing. Hum. Factors Ergon. Manuf..

[18] Yung M., Kolus A., Wells R., Neumann W.P. (2020). Examining the fatigue-quality relationship in manufacturing. Appl. Ergon..

[19] Lind C.M., Rose L.M. (2016). Shifting to proactive risk management: Risk communication using the RAMP tool. Agron. Res..

[20] Falck A.-C., Örtengren R., Rosenqvist M., Söderberg R. (2017). Proactive assessment of basic complexity in manual assembly: Development of a tool to predict and control operator-induced quality errors. Int. J. Prod. Res..

[21] Falck A.-C., Rosenqvist M. (2012). What are the obstacles and needs of proactive ergonomics measures at early product development stages?—An interview study in five Swedish companies. Int. J. Ind. Ergon..

[22] Cantley L.F., Taiwo O.A., Galusha D., Barbour R., Slade M.D., Tessier-Sherman B., Cullen M.R. (2014). Effect of systematic ergonomic hazard identification and control implementation on musculoskeletal disorder and injury risk. Scand. J. Work Environ. Health.

[23] Carrivick P.J.W., Lee A.H., Yau K.K.W. (2002). Effectiveness of a participatory workplace risk assessment team in reducing the risk and severity of musculoskeletal injury. J. Occup. Health.

[24] Lowe B.D., Dempsey P.G., Jones E.M. (2019). Ergonomics assessment methods used by ergonomics professionals. Appl. Ergon..

[25] Diego-Mas J.A., Poveda-Bautista R., Garzon-Leal D.C. (2015). Influences on the use of observational methods by practitioners when identifying risk factors in physical work. Ergonomics.

[26] Rose L.M., Eklund J., Nord Nilsson L., Barman L., Lind C.M. (2020). The RAMP package for MSD risk management in manual handling—A freely accessible tool, with website and training courses. Appl. Ergon..

[27] Lind C.M., Forsman M., Rose L.M. (2020). Development and evaluation of RAMP II—A practitioner’s tool for assessing musculoskeletal disorder risk factors in industrial manual handling. Ergonomics.

[28] Takala E.P., Pehkonen I., Forsman M., Hansson G.A., Mathiassen S.E., Neumann W.P., Sjogaard G., Veiersted K.B., Westgaard R.H., Winkel J. (2010). Systematic evaluation of observational methods assessing biomechanical exposures at work. Scand. J. Work Environ. Health.

[29] Rhén I.-M., Forsman M. (2020). Inter- and intra-rater reliability of the OCRA checklist method in video-recorded manual work tasks. Appl. Ergon..

[30] Trask C., Mathiassen S.E., Wahlström J., Forsman M. (2014). Cost-efficient assessment of biomechanical exposure in occupational groups, exemplified by posture observation and inclinometry. Scand. J. Work Environ. Health.

[31] Forsman M., Lind C.M., Vega-Barbas M., Seoane F. (2019). The Need for Practical and Reliable Risk Assessment Methods for Prevention of Musculoskeletal Disorders. Transforming Ergonomics with Personalized Health and Intelligent Workplaces.

[32] Carnevale A., Longo U.G., Schena E., Massaroni C., Lo Presti D., Berton A., Candela V., Denaro V. (2019). Wearable systems for shoulder kinematics assessment: A systematic review. BMC Musculoskelet. Disord..

[33] Lo Presti D., Carnevale A., D’Abbraccio J., Massari L., Massaroni C., Sabbadini R., Zaltieri M., Di Tocco J., Bravi M., Miccinilli S. (2020). A multi-parametric wearable system to monitor neck movements and respiratory frequency of computer workers. Sensors.

[34] Iosa M., Picerno P., Paolucci S., Morone G. (2016). Wearable inertial sensors for human movement analysis. Expert Rev. Med. Devices.

[35] Fong D.T., Chan Y.Y. (2010). The use of wearable inertial motion sensors in human lower limb biomechanics studies: A systematic review. Sensors.

[36] Poitras I., Dupuis F., Bielmann M., Campeau-Lecours A., Mercier C., Bouyer L.J., Roy J.S. (2019). Validity and reliability of wearable sensors for joint angle estimation: A systematic review. Sensors.

[37] Bark K., Hyman E., Tan F., Cha E., Jax S.A., Buxbaum L.J., Kuchenbecker K.J. (2015). Effects of vibrotactile feedback on human learning of arm motions. IEEE Trans. Neural. Syst. Rehabil. Eng..

[38] Robert-Lachaine X., Mecheri H., Larue C., Plamondon A. (2017). Accuracy and repeatability of single-pose calibration of inertial measurement units for whole-body motion analysis. Gait Posture.

[39] Ray S.J., Teizer J. (2012). Real-time construction worker posture analysis for ergonomics training. Adv. Eng. Inform..

[40] Ying Z., Morrell J.B. A vibrotactile feedback approach to posture guidance. Proceedings of the IEEE Haptic Interfaces for Virtual Environment and Teleoperator Systems (HAPTICS).

[41] Sigrist R., Rauter G., Riener R., Wolf P. (2013). Augmented visual, auditory, haptic, and multimodal feedback in motor learning: A review. Psychon. Bull. Rev..

[42] Esfahani M.I.M., Nussbaum M.A., Kong Z.Y. (2019). Using a smart textile system for classifying occupational manual material handling tasks: Evidence from lab-based simulations. Ergonomics.

[43] Mokhlespour Esfahani M.I., Nussbaum M.A. (2019). Classifying diverse physical activities using “Smart Garments”. Sensors.

[44] Yang L., Lu K., Diaz-Olivares J.A., Seoane F., Lindecrantz K., Forsman M., Abtahi F., Eklund J.A.E. (2018). Towards smart work clothing for automatic risk assessment of physical workload. IEEE Access.

[45] Vignais N., Miezal M., Bleser G., Mura K., Gorecky D., Marin F. (2013). Innovative system for real-time ergonomic feedback in industrial manufacturing. Appl. Ergon..

[46] Vignais N., Bernard F., Touvenot G., Sagot J.-C. (2017). Physical risk factors identification based on body sensor network combined to videotaping. Appl. Ergon..

[47] Lind C.M., Sandsjö L., Mahdavian N., Högberg D., Hanson L., Diaz Olivares J.A., Yang L., Forsman M., Ahram T., Karwowski W., Taiar R. (2019). Prevention of Work Related Musculoskeletal Disorders Using Smart Workwear—The Smart Workwear Consortium. Human Systems Engineering and Design.

[48] Peppoloni L., Filippeschi A., Ruffaldi E., Avizzano C.A. (2016). A novel wearable system for the online assessment of risk for biomechanical load in repetitive efforts. Int. J. Ind. Ergon..

[49] Moore J.S., Garg A. (1995). The strain index: A proposed method to analyze jobs for risk of distal upper extremity disorders. Am. Ind. Hyg. Assoc. J..

[50] Diego-Mas J.A., Alcaide-Marzal J. (2014). Using Kinect™ sensor in observational methods for assessing postures at work. Appl. Ergon..

[51] Karhu O., Kansi P., Kuorinka I. (1977). Correcting working postures in industry: A practical method for analysis. Appl. Ergon..

[52] Battini D., Persona A., Sgarbossa F. (2014). Innovative real-time system to integrate ergonomic evaluations into warehouse design and management. Comput. Ind. Eng..

[53] McAtamney L., Corlett E.N. (1993). RULA: A survey method for the investigation of work-related upper limb disorders. Appl. Ergon..

[54] Manghisi V.M., Uva A.E., Fiorentino M., Bevilacqua V., Trotta G.F., Monno G. (2016). Real time RULA assessment using Kinect v2 sensor. Appl. Ergon..

[55] Patrizi A., Pennestrì E., Valentini P.P. (2016). Comparison between low-cost marker-less and high-end marker-based motion capture systems for the computer-aided assessment of working ergonomics. Ergonomics.

[56] Waters T.R., Putz-Anderson V., Garg A., Fine L.J. (1993). Revised NIOSH equation for the design and evaluation of manual lifting tasks. Ergonomics.

[57] Alberto R., Draicchio F., Varrecchia T., Silvetti A., Iavicoli S. (2018). Wearable monitoring devices for biomechanical risk assessment at work: Current status and future challenges-a systematic review. Int. J. Environ. Res. Public Health.

[58] Verbeek J.H., Martimo K.P., Karppinen J., Kuijer P.P., Viikari-Juntura E., Takala E.P. (2011). Manual material handling advice and assistive devices for preventing and treating back pain in workers. Cochrane Database Syst. Rev..

[59] Hogan D.A., Greiner B.A., O’Sullivan L. (2014). The effect of manual handling training on achieving training transfer, employee’s behaviour change and subsequent reduction of work-related musculoskeletal disorders: A systematic review. Ergonomics.

[60] Clemes S.A., Haslam C.O., Haslam R.A. (2010). What constitutes effective manual handling training? A systematic review. Occup. Med..

[61] Doss R., Robathan J., Abdel-Malek D., Holmes M.W.R. (2018). Posture Coaching and Feedback during Patient Handling in a Student Nurse Population. IISE Trans. Occup. Ergon. Hum. Factors.

[62] Agruss C.D., Williams K., Fathallah F.A. (2004). The effect of feedback training on lumbosacral compression during simulated occupational lifting. Ergonomics.

[63] Madeleine P., Vedsted P., Blangsted A.K., Sjøgaard G., Søgaard K. (2006). Effects of electromyographic and mechanomyographic biofeedback on upper trapezius muscle activity during standardized computer work. Ergonomics.

[64] Bazazan A., Dianat I., Feizollahi N., Mombeini Z., Shirazi A.M., Castellucci H.I. (2019). Effect of a posture correction-based intervention on musculoskeletal symptoms and fatigue among control room operators. Appl. Ergon..

[65] Umek A., Kos A. (2018). Smart equipment design challenges for real-time feedback support in sport. Facta Univ. Ser. Mech. Eng..

[66] Kos M., Kramberger I. Tennis stroke consistency analysis using miniature wearable IMU. Proceedings of the IEEE Systems, Signals and Image Processing (IWSSIP).

[67] Zhang X., Shan G., Wang Y., Wan B., Li H. (2019). Wearables, biomechanical feedback, and human motor-skills’ learning & optimization. Appl. Sci..

[68] Demircan E. (2020). A pilot study on locomotion training via biomechanical models and a wearable haptic feedback system. Robomech. J..

[69] Lind C.M., Yang L., Abtahi F., Hanson L., Lindecrantz K., Lu K., Forsman M., Eklund J. (2020). Reducing postural load in order picking through a smart workwear system using real-time vibrotactile feedback. Appl. Ergon..

[70] Vega-Barbas M., Diaz-Olivares J.A., Lu K., Forsman M., Seoane F., Abtahi F. (2019). P-Ergonomics Platform: Toward precise, pervasive, and personalized ergonomics using wearable sensors and edge computing. Sensors.

[71] Mahdavian N., Lind C.M., Antonio Diaz Olivares J., Iriondo Pascual A., Högberg D., Brolin E., Yang L., Forsman M., Hanson L., Thorvald P., Case K. (2018). Effect of Giving Feedback on Postural Working Techniques. Advances in Manufacturing Technology XXXII.

[72] Ribeiro D.C., Sole G., Abbott J.H., Milosavljevic S. (2011). Extrinsic feedback and management of low back pain: A critical review of the literature. Man. Ther..

[73] Korshoj M., Skotte J.H., Christiansen C.S., Mortensen P., Kristiansen J., Hanisch C., Ingebrigtsen J., Holtermann A. (2014). Validity of the Acti4 software using ActiGraph GT3X+accelerometer for recording of arm and upper body inclination in simulated work tasks. Ergonomics.

[74] Jackson J.A., Mathiassen S.E., Wahlström J., Liv P., Forsman M. (2015). Is what you see what you get? Standard inclinometry of set upper arm elevation angles. Appl. Ergon..

[75] Hansson G.Å., Arvidsson I., Ohlsson K., Nordander C., Mathiassen S.E., Skerfving S., Balogh I. (2006). Precision of measurements of physical workload during standardised manual handling. Part II: Inclinometry of head, upper back, neck and upper arms. J. Electromyogr. Kinesiol..

[76] Kim A., Golnaraghi M.F. A quaternion-based orientation estimation algorithm using an inertial measurement unit. Proceedings of the IEEE Position Location and Navigation Symposium (PLANS).

[77] Arvidsson I., Dahlqvist C., Enquist H., Nordander C. (2017). Action Levels for Prevention of Work Related Musculoskeletal Disorders.

[78] Erlandsson A. (2002). En Utredning om Brevbärarpersonalens Arbetsförhållanden och Införandet av Bästa Metod [An Investigation into the Working Conditions of the Mail-Carrier Staff and the Introduction of Best Method].

[79] Borg G. (1998). Borg’s Perceived Exertion and Pain Scales.

[80] Kuorinka I., Jonsson B., Kilbom A., Vinterberg H., Biering-Sørensen F., Andersson G., Jørgensen K. (1987). Standardised Nordic questionnaires for the analysis of musculoskeletal symptoms. Appl. Ergon..

[81] Ciriello V.M., Maikala R.V., Dempsey P.G., O’Brien N.V. (2010). Psychophysically determined forces of dynamic pushing for female industrial workers: Comparison of two apparatuses. Appl. Ergon..

[82] Caldwell L.S., Chaffin D.B., Dukes-Dobos F.N., Kroemer K.H., Laubach L.L., Snook S.H., Wasserman D.E. (1974). A proposed standard procedure for static muscle strength testing. Am. Ind. Hyg. Assoc. J..

[83] Pheasant S., Haslegrave C.M. (2006). Bodyspace: Anthropometry, Ergonomics and the Design of Work.

[84] Kvale S., Brinkmann S., Torhell S.-E. (2014). Den Kvalitativa Forskningsintervjun [Qualitative Interviewing].

[85] Spook S.M., Koolhaas W., Bultmann U., Brouwer S. (2019). Implementing sensor technology applications for workplace health promotion: A needs assessment among workers with physically demanding work. BMC Public Health.

[86] Lind C.M., Rhen I.M., Forsman M. (2020). Bias and repeatability of standard calibration postures for inclinometry of the upper arms and trunk.

[87] Robert-Lachaine X., Mecheri H., Larue C., Plamondon A. (2017). Effect of local magnetic field disturbances on inertial measurement units accuracy. Appl. Ergon..

[88] Buchanan J.J., Wang C. (2012). Overcoming the guidance effect in motor skill learning: Feedback all the time can be beneficial. Exp. Brain Res..

[89] Lam C.F., DeRue D.S., Karam E.P., Hollenbeck J.R. (2011). The impact of feedback frequency on learning and task performance: Challenging the “more is better” assumption. Organ. Behav. Hum. Decis. Process..

[90] Patterson J.T., Carter M.J., Hansen S. (2013). Self-controlled KR schedules: Does repetition order matter?. Hum. Mov. Sci..

[91] Gerard M.J., Armstrong T.J., Rempel D.A., Woolley C. (2002). Short term and long term effects of enhanced auditory feedback on typing force, EMG, and comfort while typing. Appl. Ergon..

